# Hydrogenative Ring-Rearrangement of Furfural to Cyclopentanone over Pd/UiO-66-NO_2_ with Tunable Missing-Linker Defects

**DOI:** 10.3390/molecules26195736

**Published:** 2021-09-22

**Authors:** Chunhua Wang, Zhiquan Yu, Yuhao Yang, Zhichao Sun, Yao Wang, Chuan Shi, Ying-Ya Liu, Anjie Wang, Karen Leus, Pascal Van Der Voort

**Affiliations:** 1 State Key Laboratory of Fine Chemicals, School of Chemical Engineering, Dalian University of Technology, Dalian 116024, China; 274638799@mail.dlut.edu.cn (C.W.); yuzhiquan@dlut.edu.cn (Z.Y.); yyh917401123@163.com (Y.Y.); sunzhichao@dlut.edu.cn (Z.S.); wangyao@dlut.edu.cn (Y.W.); chuanshi@dlut.edu.cn (C.S.); 2 Liaoning Key Laboratory of Petrochemical Technology and Equipment, Dalian University of Technology, Dalian 116012, China; 3 Department of Chemistry, Ghent University, Krijgslaan 281-S3, 9000 Ghent, Belgium; karen.leus@ugent.be (K.L.); pascal.vandervoort@ugent.be (P.V.D.V.)

**Keywords:** Pd/UiO-66-NO_2_, furfural, hydrogenative ring-rearrangement, cyclopentanone, double-solvent method

## Abstract

Upgrading furfural (FAL) to cyclopentanone (CPO) is of great importance for the synthesis of high-value chemicals and biomass utilization. The hydrogenative ring-rearrangement of FAL is catalyzed by metal-acid bifunctional catalysts. The Lewis acidity is a key factor in promoting the rearrangement of furan rings and achieving a high selectivity to CPO. In this work, highly dispersed Pd nanoparticles were successfully encapsulated into the cavities of a Zr based MOF, UiO-66-NO_2_, by impregnation using a double-solvent method (DSM) followed by H_2_ reduction. The obtained Pd/UiO-66-NO_2_ catalyst showed a significantly better catalytic performance in the aforementioned reaction than the Pd/UiO-66 catalyst due to the higher Lewis acidity of the support. Moreover, by using a thermal treatment. The Lewis acidity can be further increased through the creating of missing-linker defects. The resulting defective Pd/UiO-66-NO_2_ exhibited the highest CPO selectivity and FAL conversion of 96.6% and 98.9%, respectively. In addition, the catalyst was able to maintain a high activity and stability after four consecutive runs. The current study not only provides an efficient catalytic reaction system for the hydrogenative ring-rearrangement of furfural to cyclopentanone but also emphasizes the importance of defect sites.

## 1. Introduction

The value-added conversion of biomass at high efficiency has become increasingly attractive for the sustainable production of clean fuels and chemicals [1,2]. Within this regard, furfural (FAL) is an important biomass-derived compound from xylose and hemicellulose [3,4]. Due to the presence of a rich unsaturated bond in the structure, FAL can be converted to various value-added chemicals such as furfuryl alcohol (FOL), tetrahydrofurfuryl alcohol (THFOL), 2-methyl furan (2-MF), furan, cyclopentanone (CPO) and cyclopentanol (CPL) [3,5,6].

CPO is an important fine chemical intermediate, which can be used to synthesize medicines [7], fragrance chemicals [8], rubber chemicals [9], and pesticides [10]. In addition, it is widely used in the electronic industry as a solvent [11]. Conventionally, CPO is synthesized by the pyrolysis of adipic acid or by the oxidation of cyclopentene [12]. However, the raw materials for the conventional production come from unsustainable fossil fuels, and the processes are not energy-effective. Recently, a new synthesis pathway was proposed for the production of CPO from FAL. The hydrogenative ring-rearrangement of FAL to CPO is a multi-step process, including C=O hydrogenation, furan ring-rearrangement, dehydration, and C=C hydrogenation [13]. In general, the hydrogenative ring-rearrangement of FAL reaction is carried out in an aqueous phase over metal-acid bifunctional catalysts [13]. Various noble metals (Pd, Pt, Ru) and transition metals (Cu, Ni, Co) based catalysts have been demonstrated to be able to catalyze the hydrogenative ring-rearrangement of FAL to CPO [14,15,16,17]. Among them, Pd-based catalysts (Pd/C [18], Pd/f-SiO_2_ [19], Pd/Y_2_(Sn_0.65_Al_0.35_)_2_O_7−δ_/Al_2_O_3_ [20], Pd/Y_2_(Sn_7_Al_0.3_)_2_O_7−δ_ [21], Pd/FeZn-DMC [22]) showed a good performance under mild conditions. It has been shown that Lewis acidity is a key factor in promoting the rearrangement of furan rings and achieving a high selectivity to CPO, whereas the Brønsted acid sites promote the polymerization to produce undesired products [23]. Hence, designing a Pd-based catalyst with suitable Lewis acidity is important to achieve an efficient production of CPO. Metal-organic frameworks (MOFs) are promising candidates to fulfill these criteria, because of their high tunability which allows an easy adjustment of the acidity in the structure [24].

Recently, MOFs-supported Pd nanoparticles showed a good catalytic performance in the hydrogenative ring-rearrangement of FAL. MOFs possess large surface areas and contain a certain amount of Lewis acid sites at the unsaturated metal centers. Li et al. investigated the catalytic performance of Pd/Fe-MIL-100 in the hydrogenative ring-rearrangement of FAL to CPO. The authors observed a FAL conversion of 99.0% and a CPO yield of 92.2%. It was proposed that the Lewis acid sites provided by the unsaturated metal sites in Fe-MIL-100 played a crucial role in the furan ring-rearrangement to CPO [14]. Within the family of MOF materials, UiO-66, is a highly stable MOF [25]. The MOF is built up from [Zr_6_O_4_(OH)_4_(CO_2_)_12_] clusters linked with 1,4-benzenedicarboxylate ligands, featuring a porous cubic architecture. The framework consists of tetrahedral and octahedral cages with dimensions close to 8 and 12 Å, respectively. These cages are interconnected through narrow triangular windows with window sizes close to 6 Å. Our earlier work has demonstrated that Pd/UiO-66 is a good hydrogenation catalyst in FAL hydrogenation to THFOL (100% conversion and selectivity) in an aqueous phase at 60 °C [26]. However, Wang et al. showed that Pd/UiO-66 was not effective to catalyze the selective transformation of FAL to CPO as only a selectivity of 76% to CPO was noted [27]. This might be due to the weak Lewis acidity of the framework. Several strategies can effectively enhance the Lewis acidity of the MOF framework. For example, the defect-engineering, which is widely used for zeolites [28], is an effective approach to increase the amount of Lewis acid sites in the MOF framework. The thermal treatment will result in the formation of defects in the MOF structure and will enhance the specific surface area, pore size, and Lewis acid sites [29,30]. The tunable features of MOFs ensure the encapsulation and confinement of metal nanoparticles by forming a weak bond between the metal precursor and unsaturated metal center, thus improving the metal dispersion and preventing metal leaching [31].

In the present work, we report on the catalytic hydrogenative ring-rearrangement of FAL to CPO in an aqueous phase using Pd/UiO-66-NO_2_ as the catalyst. The Lewis acidity of UiO-66-NO_2_ was easily enhanced by using a thermal treatment. Pd nanoparticles were encapsulated into the cavities of the framework by using a double solvent method (DSM) followed by H_2_ reduction. Various experimental parameters, such as the thermal treatment time, the concentration of FAL, reaction temperature, and hydrogen pressure were investigated, to achieve a high conversion of FAL and high selectivity to CPO.

## 2. Results and Discussion

### 2.1. Catalyst Characterization

**Figure 1:**
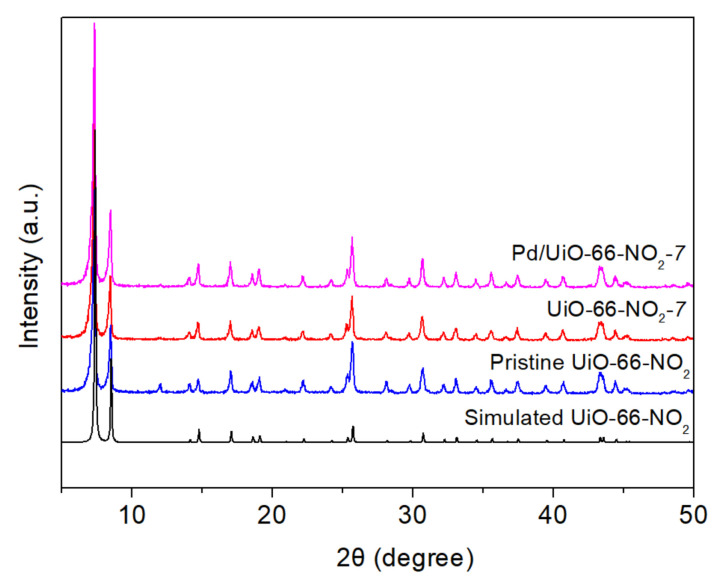
XRD patterns of the simulated UiO-66-NO_2_, pristine UiO-66-NO_2_, UiO-66-NO_2_-*7*, and Pd/UiO-66-NO_2_-*7*.

The XRD patterns of the UiO-66-NO_2_ material are presented in Figure 1. The diffraction pattern of the pristine UiO-66-NO_2_ matched well with the reported simulated pattern [32]. The comparison of the XRD data of the UiO-66-NO_2_ before and after the thermal treatment is shown in Figure S1a (UiO-66-NO_2_-*t*, where *t* stands for the thermal treatment time at 300 °C and the time unit is hours). Upon the thermal treatment, the diffraction peak at 12.0° nearly disappeared, indicating a slight change in the structural regularity in the short-range order during the dehydroxylation process [32]. However, the other diffraction peaks correspond well with the pristine UiO-66-NO_2_, indicating that the topology of the parent framework was stable upon the thermal treatment. The Pd/UiO-66-NO_2_-*7* material was prepared by introducing a Pd precursor into the MOF by DSM and subsequent H_2_ reduction. As shown in Figure 1, the XRD pattern of the Pd encapsulated MOF is in line with the parent MOF, indicating that the encapsulation of Pd did not change the structure of the parent UiO-66-NO_2_-*7*. No characteristic diffractions for the Pd species were observed at a Pd loading of 3 wt%. Similar results were obtained for UiO-66 and Pd/UiO-66 (see Figure S1b).

The SEM images of the UiO-66 based materials are depicted in Figure S2. All the materials have similar morphology with cubic-shaped small crystallites (100–120 nm). As expected, after the thermal treatment at 300 °C, the size and morphology of the UiO-66-NO_2_-*t* did not change up to a treatment time of 24 h (Figure S2b–g). Moreover, no significant change in the crystallite morphology was observed after the encapsulation of the Pd nanoparticles (Figure S2j). Similar results were also observed on UiO-66 (Figure S2h,i) and Pd/UiO-66-*7* (Figure S2k).

**Figure 2:**
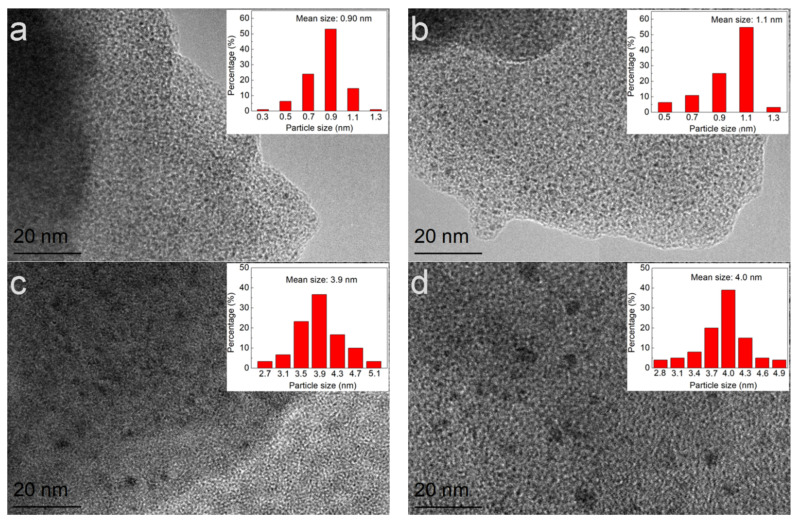
TEM images and particle size distributions of Pd: (**a**) Pd/UiO-66-NO_2_-*7*; (**b**) Pd/UiO-66-*7*; (**c**) Pd/UiO-66-NO_2_-*7*(IMP); (**d**) Pd/UiO-66-*7*(IMP).

The structural features, as well as the dispersion of the Pd nanoparticles, were analyzed by means of TEM (Figure 2). The average size of the Pd nanoparticles in Pd/UiO-66-NO_2_-*7* and Pd/UiO-66-*7* were 0.9 and 1.1 nm, respectively. Or in other words, the nanoparticles are smaller than the pore size of UiO-66-NO_2_, indicating that the Pd particles are likely embedded in the pores of UiO-66-NO_2_ without any obvious agglomeration. The average particle size of Pd/UiO-66-NO_2_-*7*(IMP) and Pd/UiO-66-*7*(IMP) were 3.9 and 4.0 nm, respectively. As the Pd particles are larger than the pore size of the parent MOF, they are mainly deposited on the external surface. In conclusion, the DSM is an effective and facile method of confining the Pd nanoparticles in the pores of UiO-66-based MOFs.

The BET-specific surface area of all the activated UiO-66-NO_2_-*t* (*t* = *1*, *3*, *5*, *7*, *12*, *24*) and Pd/MOFs samples were determined from the N_2_ adsorption/desorption isotherms at −196 °C. As shown in Figure S3, each MOF material exhibits a type I isotherm, characteristic of microporous materials. Both the BET surface area (*S*_BET_) and pore volume are summarized in Table 1. The *S*_BET_ of the pristine UiO-66-NO_2_ is 735 m^2^·g^−1^. After the thermal treatment at 300 °C, both the *S*_BET_ and the pore volume of the UiO-66-NO_2_-*t* increased with the thermal treatment time (Table S1), probably due to the missing linker caused by the thermal treatment, which is consistent with the literature report [33]. After the encapsulation of Pd nanoparticles, both the *S*_BET_ and the pore volume of Pd/UiO-66-NO_2_-*7* (*S*_BET_ = 563 m^2^·g^−1^) decreased in comparison to that of UiO-66-NO_2_-*7*, which can be attributed to the partial occupation of the MOF cavities by the Pd particles. Similar results were also observed for the Pd/UiO-66-*7* material. The Pd/UiO-66-NO_2_-*7*(IMP) exhibits a lower specific surface area (*S*_BET_ = 519 m^2^·g^−1^) and a smaller pore volume compared to Pd/UiO-66-NO_2_-*7*, due to the partial blockage by Pd particles at the pore mouths of UiO-66-NO_2_-*7* [31]. The dispersion of Pd was determined by CO pulse chemisorption, and the results are listed in Table 1. It indicated that the dispersion of Pd in Pd/UiO-66-NO_2_-*7*, Pd/UiO-66-*7*, and Pd/UiO-66-NO_2_-*7*(IMP) was 49.8%, 47.2%, and 31.2%, respectively. This result clearly demonstrates that the dispersion of Pd prepared by DSM is higher than by the conventional impregnation method.

**Figure 3:**
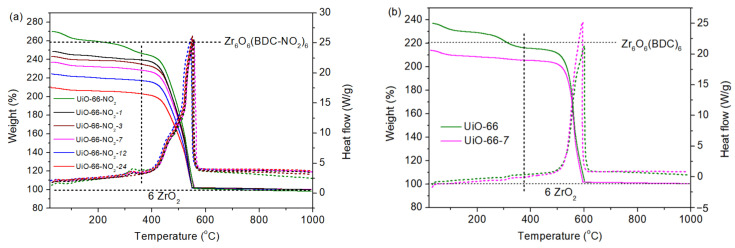
TG−DSC curves of (**a**) the UiO-66-NO_2_-*t* samples, (**b**) the UiO-66 and UiO-66-*7*.

In literature, it has been demonstrated that a thermal treatment of UiO-66 based materials can enhance the missing linker defects in the structure [34,35]. Thermogravimetric analysis (TG) is one of the most accessible methods that can provide information about these missing-linker defects in MOFs [36]. As illustrated in Figure 3, three weight loss steps were observed in the TG-DSC curves for the prepared samples. To quantify the amount of the missing linkers, the residue weight percentage was normalized to 100%. The weight losses are assigned to the following processes: (1) release of physically adsorbed water and residual solvent molecules (DMF and ethanol) below 200 °C, (2) the dehydroxylation of the Zr_6_ cornerstones at 200–355 °C, and (3) the complete decomposition of the framework at 355–550 °C. ZrO_2_ was the residue product in airflow. The ideal structural unit of UiO-66-NO_2_ is Zr_6_O_4_(OH)_4_(BDC-NO_2_)_6_, while the defect-free but dehydroxylated UiO-66-NO_2_ consists of Zr_6_O_6_(BDC-NO_2_)_6_ units. Following the method reported by Lillerud et al. [33], the TG data was further analyzed to evaluate the deficiencies of BDC-NO_2_ linkers in the samples (Figure 3a). The value at 355 °C and the final weight (100%) were correlated to the defective UiO-66-NO_2_ and the decomposition product, with a formula unit of Zr_6_O_6+x_(BDC-NO_2_)_6-x_ (in which x stands for the deficient value of BDC-NO_2_ linkers per Zr_6_ formula unit) and 6 ZrO_2_, respectively. In the ideal UiO-66-NO_2_ structure, x is zero and the theoretical weight at 355 °C of the Zr_6_O_6_(BDC-NO_2_)_6_ is 257.6%. The weight loss between the labeled “Zr_6_O_6_(BDC-NO_2_)_6_” and “6 ZrO_2_” represents the theoretical weight loss of 6 linkers. The linker deficiencies were calculated to be 0.43 and 0.21 for UiO-66-NO_2_ (Figure 3a) and UiO-66 (Figure 3b), respectively. The TG curves of the observed weight values at 355 °C for all the UiO-66-NO_2_-*t* samples were smaller than 257.6%, demonstrating that there were more missing linker defects after the thermal treatment. Moreover, the deficiencies of the BDC-NO_2_ linkers increased with enhancing the thermal treatment time, and the amounts of the missing-linker in UiO-66-NO_2_-*1*, UiO-66-NO_2_-*3*, UiO-66-NO_2_-*5*, UiO-66-NO_2_-*7*, UiO-66-NO_2_-*12*, and UiO-66-NO_2_-*24* were calculated to be 0.71, 0.82, 0.99, 1.50, and 1.90, respectively. For UiO-66-*7*, the missing linkers defects were 0.75 after the thermal treatment at 300 °C for 7 h.

**Figure 4:**
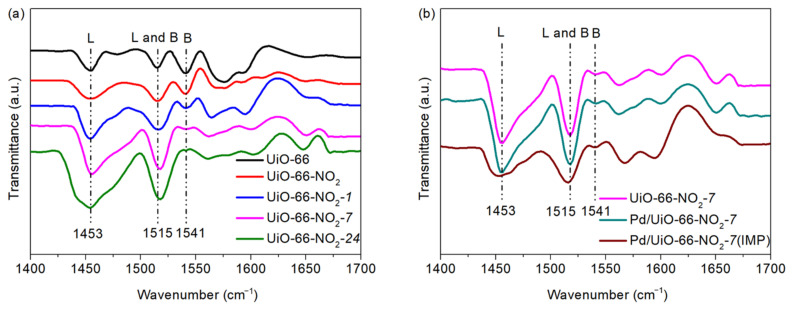
FT−IR spectra of adsorbed pyridine on (**a**) UiO-66 and UiO-66-NO_2_-*t*; (**b**) UiO-66-NO_2_-*7*, Pd/UiO-66-NO_2_-*7*, and Pd/UiO-66-NO_2_-*7*(IMP).

The FT-IR of adsorbed pyridine was carried out to examine the surface acidity of UiO-66-NO_2_ by using pyridine as a probe molecule. In the spectrum of UiO-66-NO_2_ (Figure 4), the bands at 1453 cm^−1^ and 1541 cm^−1^ were ascribed to Lewis-bonded pyridine and Brønsted-bonded pyridine, respectively, while the vibration at 1515 cm^−1^ can be assigned to pyridine associated with Brønsted and Lewis acid sites. The Lewis acidity of UiO-66-NO_2_ might originate from the accessible metal Zr sites [37], as well as from the defects due to the missing organic linkers between the Zr_6_ clusters created during the synthesis [32]. The Brønsted acidity, on the other hand, originates from the protonic organic linkers in UiO-66-NO_2_. The same three bands were also observed in the FT-IR spectrum of UiO-66. By looking at the relative intensities of the bands it was shown that UiO-66 (L/B:1.3) exhibited a weaker Lewis acidity compared to UiO-66-NO_2_ (L/B:2.1). Or in other words, the introduction of the nitro functional group into the UiO-66 framework enhanced the Lewis acidity, due to the electronic effects of linker substitution [38]. After the thermal treatment at 300 °C, UiO-66-NO_2_-*t* exhibited a significantly increased Lewis acidity upon increasing the thermal treatment time (Figure 4a). Also in the FT-IR spectrum for the Pd/UiO-66-NO_2_-*7* material (Figure 4b), the three bands were present, without any notable changes in the peak intensity, suggesting that the acidic sites were well preserved after the encapsulation of the Pd nanoparticles. In contrast, when Pd nanoparticles were introduced into the UiO-66-NO_2_-*7* material by using the impregnation method (Pd/UiO-66-NO_2_-*7*(IMP)), an obvious decrease in the intensity of the band at 1453 cm^−1^ assigned to Lewis acid sites was observed. This is probably due to the fact that the Pd nanoparticles occupy partially the unsaturated sites of Zr^4+^, which hinders the adsorption of pyridine on these sites.

### 2.2. Hydrogenative Ring-Rearrangement of Furfural

**Scheme 1:**
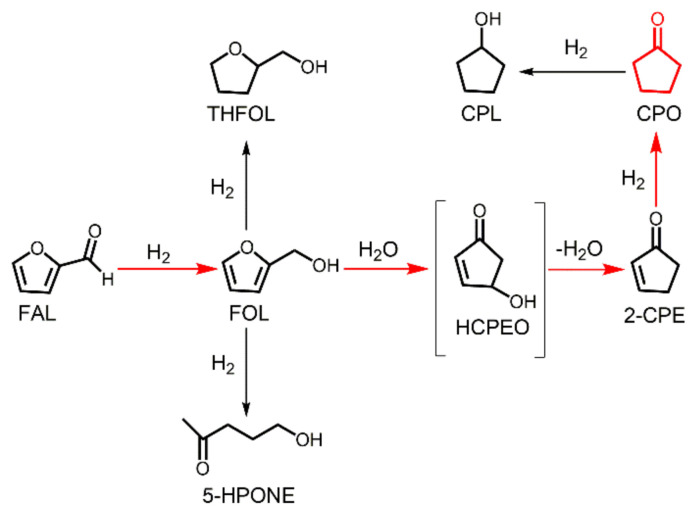
Possible reaction pathways for the hydrogenative ring-rearrangement of furfural to cyclopentanone [40].

In Scheme 1, the possible reaction pathways from FAL to CPO are depicted as well as the possible by-products formed during the hydrogenation reaction based on the literature and our experimental data [39,40]. The hydrogenative ring-rearrangement of FAL takes place in the following steps. In a first step, the C=O group of FAL is hydrogenated to FOL. Secondly, the Lewis acid-catalyzed rearrangement of the furan ring from FOL results in an intermediate product 4-hydroxy-cyclopentenone (HCPEO) in the presence of water, then HCPEO is further converted into 2-cyclopentenone (2-CPE) by dehydration. In the final step, 2-CPE is further hydrogenated to the target product CPO. However, deep hydrogenation can occur, in which CPO and FOL are converted to CPL and THFOL, respectively. The presence of strong Lewis acid or Brønsted acid sites may catalyze the polymerization, resulting in a loss of the carbon balance and deactivation of the catalyst. Besides this, also ring-opening products can be generated, such as 5-hydroxy-2-pentanone (5-HPONE).

**Figure 5:**
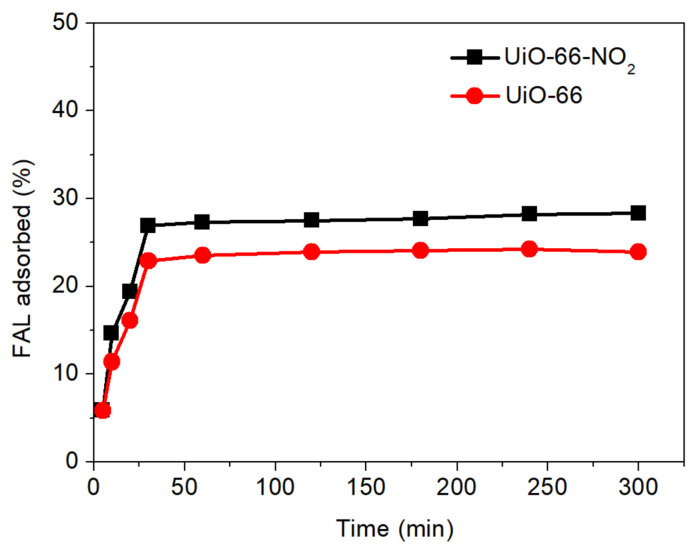
Variation of adsorbed FAL with time in static adsorption.

The adsorption of FAL on the support is a prerequisite in the hydrogenation process. Figure 5 illustrated that UiO-66-NO_2_ shows a higher adsorption uptake of FAL than UiO-66. This higher adsorption capacity of UiO-66-NO_2_ might be the result of the presence of the NO_2_ functional groups in the MOF structure. In previous reports, it was already shown that the adsorption of FAL is enhanced by the presence of stronger acid sites on the support [41,42].

The hydrogenative ring-rearrangement of FAL requires both the Lewis acid and metal center as the catalytic sites. The Lewis acid site catalyzes the ring-rearrangement, while Pd is responsible for the hydrogenation step. The catalytic performance of Pd supported MOF catalysts with different Pd particle sizes and metal dispersion are shown in Table 2. In the control runs using the parent UiO-66-NO_2_ or UiO-66 as a catalyst, no conversion of FAL was obtained in the absence of Pd. Since the first step of the reaction is a hydrogenation, the reaction was unable to take place, without the participation of Pd.

When Pd/UiO-66 was used as a catalyst, 89.2% FAL conversion and a selectivity of 88.9% to CPO was achieved in 5 h at 1MPa H_2_. The main by-product was THFOL (8.3%), and trace amounts of the intermediates FOL and 2-CPE were observed. The functionalized, Pd/UiO-66-NO_2_ gave a similar product distribution compared to Pd/UiO-66, and showed a slightly higher FAL conversion (92.3%) and CPO selectivity (91.0%). In contrast, the Pd/UiO-66(IMP) and Pd/UiO-66-NO_2_(IMP) presented a lower catalytic activity, and a FAL conversion of 83.7% and 84.8% was obtained, respectively. The lower activity might be the result of the larger Pd nanoparticles and the lower metal dispersion (Figure 2, Table 1). When Pd/UiO-66(IMP) and Pd/UiO-66-NO_2_(IMP) were used as a catalyst with the same Pd loading, the CPO selectivity was only 81.4% and 85.9% respectively, and the selectivity to THFOL (9.1% and 6.9%) increased. The reason for the decreased selectivity to CPO might be that the acidity of the support was hindered to a certain extent as the Pd nanoparticles preferred to be deposited on the outer surface when using the impregnation method (Figure 2). As a result, the outstanding performance of the Pd/UiO-66-NO_2_ catalyst is probably the result of the following combined effects: (1) -NO_2_ group facilitates the adsorption of FAL, which would further increase the hydrogenation reaction rate and (2) the Lewis acid sites favor the ring-rearrangement reaction. All the catalysts prepared by the DSM exhibited better activity than those prepared by the impregnation method because the former catalysts had a higher metal dispersion and stronger Lewis acidity (see pyridine FT-IR spectra in Figure 4). The stronger Lewis acidity is beneficial for the rearrangement of FOL, thus improving the selectivity to CPO.

#### 2.2.1. Effect of Thermal Treatment Time

Based on the aforementioned investigation, it was shown that -NO_2_ functionalization has a positive effect on the CPO selectivity. To further increase the Lewis acidity of the UiO-66-NO_2_ material, a thermal treatment was performed, the stronger of Lewis acidity was confirmed by TG-DSC and Py-IR analysis (Figure 3 and Figure 4). The activity and the product selectivity of the Pd/UiO-66-NO_2_-*t* catalysts are presented in Table 3. After the thermal treatment, all the catalysts exhibited an increased catalytic performance. A maximum FAL conversion of 98.9 % was achieved when using the Pd/UiO-66-NO_2_-*5* material as a catalyst, while the Pd/UiO-66-NO_2_ exhibited a FAL conversion of 92.3%. No change in the conversion was noted upon a further increase of the thermal treatment time. In addition, the thermal treatment time also influenced the product’s selectivity. The highest CPO selectivity was obtained when using Pd/UiO-66-NO_2_-*7* (96.6%) as a catalyst. A further increase of the thermal treatment time to 12 h and 24 h resulted in reduced selectivity to CPO (92.3% and 91.6%). For the major intermediate product FOL, its selectivity decreased with increasing the Lewis acidity (by increasing the thermal treatment time). When the thermal treatment time was higher than 12 h, FOL was not anymore detected. Meanwhile, the selectivity to the hydrogenated product THFOL followed the same trend (1–7 h). When the thermal treatment time was 7 h, the selectivity to THFOL decreased to 2.5%, indicating that the higher Lewis acidity accelerated the conversion of FOL to the rearrangement product. However, at a certain point, the higher Lewis acidity also leads to the formation of some unknown products. From these experiments, it was concluded that the Pd/UiO-66-NO_2_-*7* material was the best catalyst for converting FAL to CPO. For comparison, the FAL conversion and CPO selectivity over Pd/UiO-66-*7* was much lower compared to Pd/UiO-66-NO_2_-*7*. Form literature, it is known that a stronger Lewis acidity is beneficial for the hydrogenation and rearrangement process, or in other words, it improves the selectivity to CPO [14,21,22].

#### 2.2.2. Effect of Pd Loading

**Figure 6:**
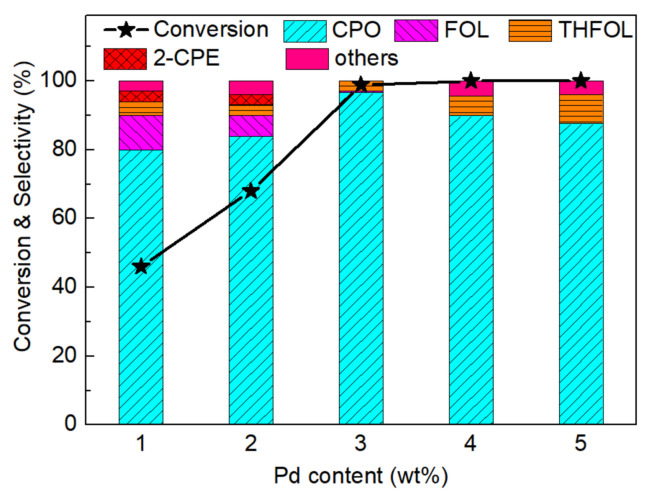
Effect of Pd loading on conversion and product selectivity in hydrogenative ring-rearrangement of FAL over Pd/UiO-66-NO_2_-*7*. Reaction conditions: catalyst (5.0 mg), H_2_O (9.9 g), 150 °C, 1.0 MPa H_2_ pressure, 5 h.

The catalytic performance of Pd/UiO-66-NO_2_-*7* using different Pd loadings was examined. As shown in Figure 6, the conversion of FAL was 46.0% with a CPO selectivity of 80.0% when the Pd loading was 1%. As the Pd loading increased to 2%, the conversion of FAL increased to 68.0% and the selectivity of CPO increased to 84.0%. The best catalytic performance was obtained with a Pd loading of 3% showing a FAL conversion of 98.9% and a 96.6% CPO selectivity. A further increase in the Pd loading resulted in a significant decrease in the selectivity to CPO due to the excessive hydrogenation of FOL to form THFOL.

#### 2.2.3. Effect of FAL Concentration

**Figure 7:**
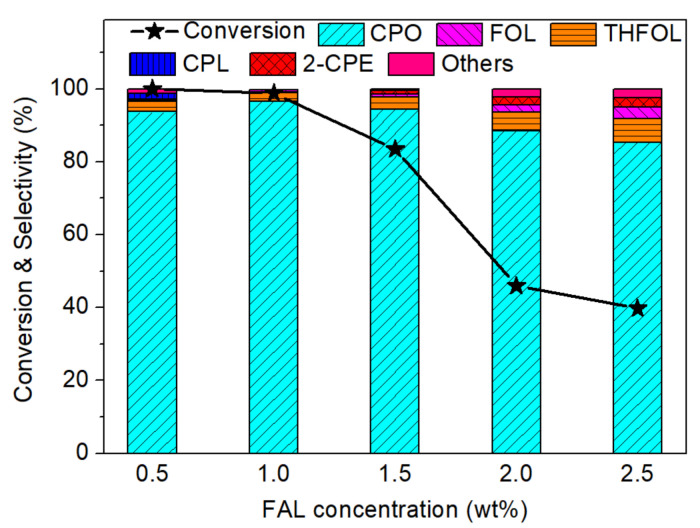
Effect of FAL concentration on conversion and product selectivity in hydrogenative ring-rearrangement of FAL over Pd/UiO-66-NO_2_-*7*. Reaction conditions: catalyst (5.0 mg), H_2_O (9.9 g), 150 °C, 1.0 MPa H_2_ pressure, 5 h.

The variation of FAL conversion and product selectivity as a function of the FAL concentration (0.5–2.5 wt%) in the hydrogenative ring-rearrangement catalyzed by Pd/UiO-66-NO_2_-*7* is presented in Figure 7. With increasing the FAL concentration in the feed, the selectivity of CPO increased initially after which is started to decrease. A maximum selectivity of 96.6% at a FAL conversion of 98.9% was obtained when the concentration of FAL was 1 wt%. A further increase in the concentration of FAL resulted in a significant decrease in both the conversion and selectivity to CPO. A high concentration of FAL in an aqueous solution likely causes a carbon loss due to the polymerization of FAL and/or the hydrogenated intermediate (FOL) [43].

#### 2.2.4. Effect of Reaction Temperature

**Figure 8:**
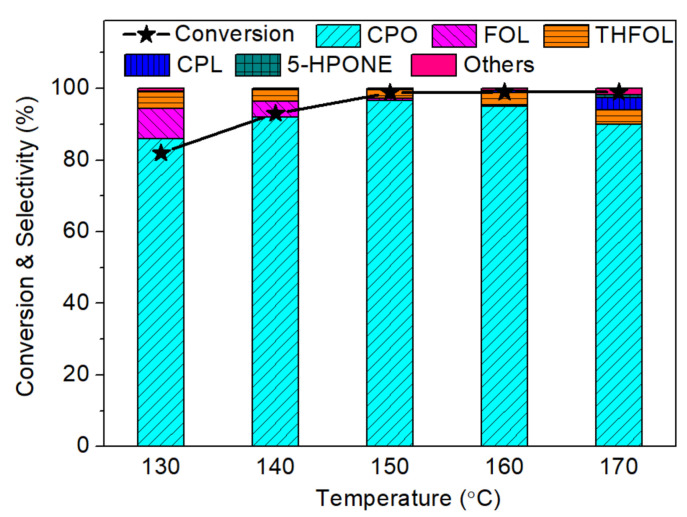
Effect of reaction temperature on conversion and product selectivity in hydrogenative ring-rearrangement of FAL over Pd/UiO-66-NO_2_-*7*. Reaction conditions: catalyst (5.0 mg), FAL (100.0 mg), H_2_O (9.9 g), 1.0 MPa H_2_ pressure, 5 h.

Figure 8 shows the effect of temperature on the conversion of FAL and product selectivity. The highest selectivity to CPO was 96.6% at a FAL conversion of 98.9% at 150 °C. At a lower temperature (130 °C), the FAL conversion and CPO selectivity was 82.0% and 86.0%, respectively. Increasing the temperature from 130 °C to 150 °C, resulted in increased conversion of FAL from 82.0% to 98.9% which leveled off at 150–170 °C. The CPO selectivity increased with increasing the reaction temperature up until 150 °C, indicating that an appropriate increase of reaction temperature would activate the furan ring to promote the generation of CPO [44]. However, the selectivity to CPO decreased with a further increase of the temperature from 150 °C to 170 °C, and the over-hydrogenated product (CPL) and ring-opening product (5-HPONE) were obtained. Besides, the undesired polymerization and coking at higher temperatures result in carbon deposition on the catalyst surface [45].

#### 2.2.5. Effect of Hydrogen Pressure

**Figure 9:**
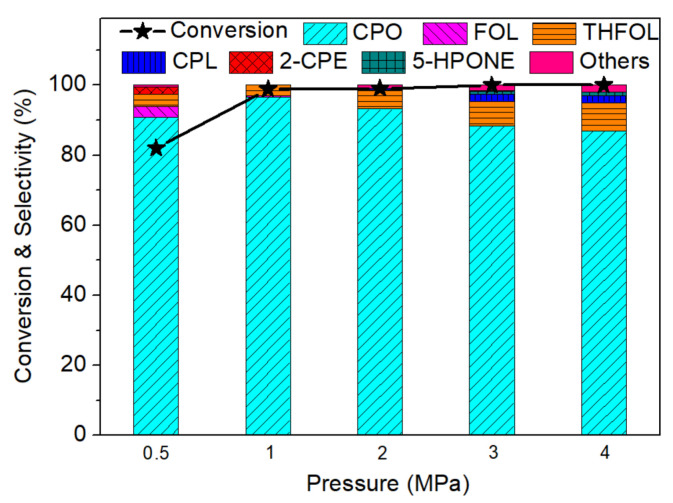
Effect of hydrogen pressure on conversion and product selectivity in hydrogenative ring-rearrangement of FAL over Pd/UiO-66-NO_2_-*7*. Reaction conditions: catalyst (5.0 mg), FAL (100.0 mg), H_2_O (9.9 g), 150 °C, 5 h.

Figure 9 depicts the influence of the hydrogen pressure on the catalytic performance of Pd/UiO-66-NO_2_-7 in the hydrogenative ring-rearrangement reaction. With increasing the hydrogen pressure from 0.5 to 1.0 MPa, a drastic increase in the conversion of FAL from 82.0% to 98.9% was observed. Moreover, the CPO selectivity increased simultaneously from 90.9% to 96.6%. At higher H_2_ pressures (2.0~4.0 MPa), FAL was completely converted but this was accompanied by a decrease in the CPO selectivity to 87.0% at 4 MPa and an increase in the furan ring hydrogenation product (THFOL: 8.0%) and over-hydrogenated product (CPL: 2.0%).

#### 2.2.6. Effect of Reaction Time

**Figure 10:**
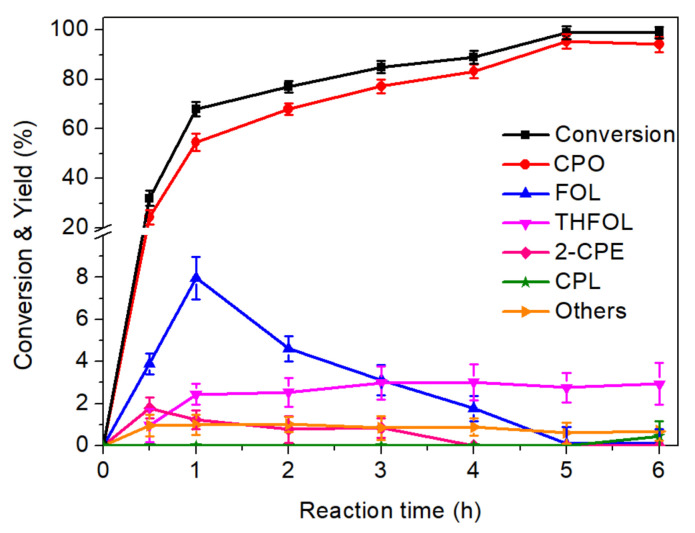
Variation of conversion and yield with reaction time in hydrogenative ring-rearrangement of FAL over Pd/UiO-66-NO_2_-*7*. Reaction conditions: catalyst (5.0 mg), FAL (100.0 mg), H_2_O (9.9 g), 150 °C, 1.0 MPa H_2_ pressure.

Figure 10 displays the FAL conversion and product yield as a function of time over Pd/UiO-66-NO_2_-*7* under the optimized reaction conditions (150 °C and 1.0 MPa H_2_). The FAL conversion steadily increased with time and reached 98.9% after 5 h. The yield of CPO passed through a maximum yield of 96.6% at the fifth hour. The yield of FOL and 2-CPE increased initially in the first hour but quickly decreased afterward, indicating that FOL and 2-CPE were the intermediate products. THFOL was formed by the hydrogenation of the furan ring of FOL, and the yield (~3.0%) did not change in the course of the reaction. Meanwhile, CPL as the over-hydrogenated product of CPO was detected at 6 h. In comparison to other supported Pd catalysts, such as Pd/C [18], Pd/f-SiO_2_ [19], and Pd/Fe-MIL-100 [14], Pd/UiO-66-NO_2_-*7* gave a higher yield of CPO, owing to the highly dispersed Pd nanoparticles and suitable Lewis acidity.

#### 2.2.7. Catalyst Stability and Recyclability

**Figure 11:**
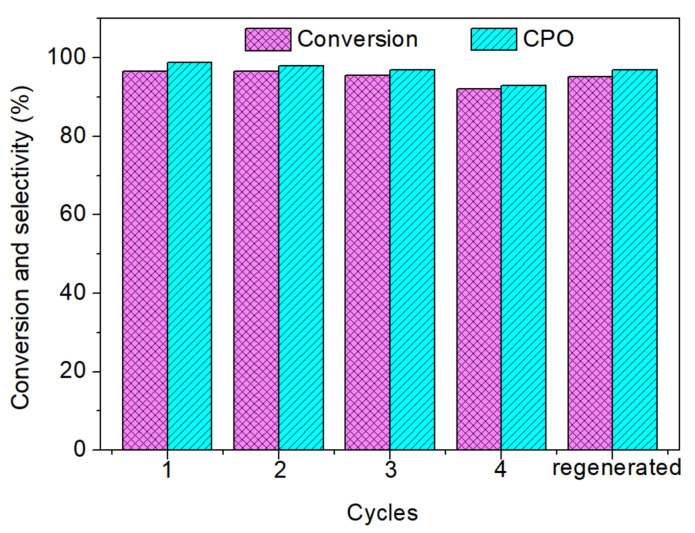
Recycling experiments of hydrogenative ring-rearrangement of FAL to CPO over Pd/UiO-66-NO_2_-*7*. Reaction conditions: catalyst (5.0 mg), FAL (100.0 mg), H_2_O (9.9 g), 150 °C, 1.0 MPa H_2_ pressure, 5 h.

**Figure 12:**
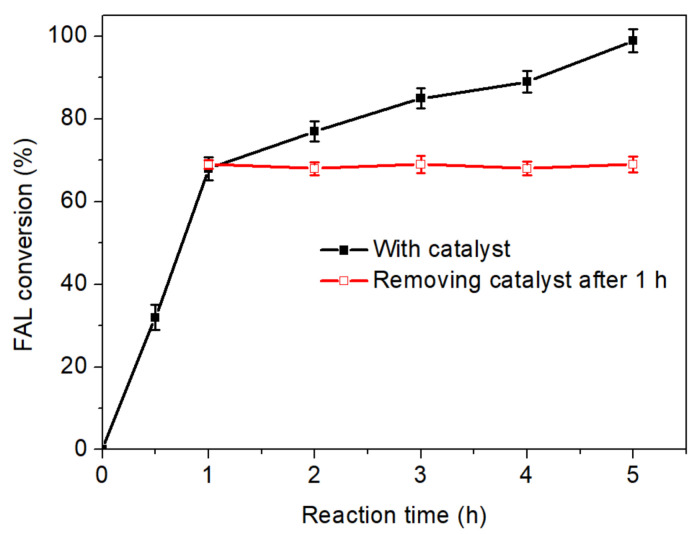
Hot filtration experiment of Pd/UiO-66-NO_2_-*7*. Reaction conditions: catalyst (5.0 mg), FAL (100.0 mg), H_2_O (9.9 g), 150 °C, 1.0 MPa H_2_ pressure.

**Figure 13:**
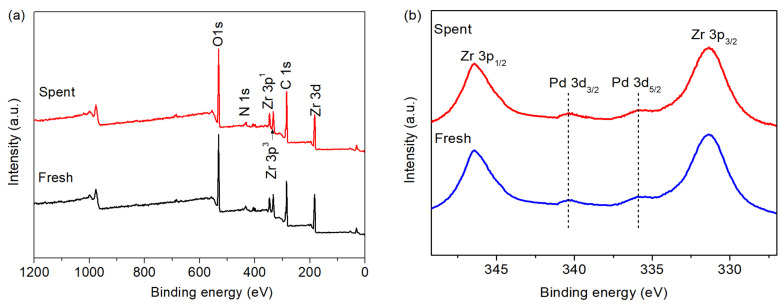
XPS spectra of Pd/UiO-66-NO_2_-*7* (**a**) survey spectrum, (**b**) high-resolution XPS spectra of Zr 3p and Pd 3d.

To evaluate the stability and recyclability of the Pd/UiO-66-NO_2_-*7* catalyst in the hydrogenative ring-rearrangement of FAL, a four-cycle experiment was performed using the optimized reaction conditions. After each reaction cycle, the catalyst was recovered by centrifugation, washed with water, acetone, and dried before use in the subsequent run. The results are illustrated in Figure 11. During the first two cycles, the FAL conversion and CPO selectivity did not change. In the third cycle, the conversion of FAL dropped slightly to 96%, and the CPO selectivity decreased to 95.6%. After the fourth cycle, the selectivity to CPO decreased from 98.9% to 93.0%, with the conversion of FAL decreasing from 96.6% to 92.0%. This clearly shows that the activity of the spent catalyst almost resumed after activating in the presence of H_2_. Another important aspect to evaluate is the leaching of Pd in the solution. Therefore, a hot-filtration experiment was performed. After 1 h, the catalyst was removed, and the solution was collected and allowed to react for another 4 h under identical conditions (Figure 12). No further conversion of FAL was detected after the removal of the catalyst, indicating that no Pd was leached during the reaction. In addition, the solution collected after the recovery of the catalyst was analyzed by means of ICP-OES. No Pd was detected in the solution which completely rules out the possibility of leaching of Pd species. In addition, the leaching of Zr species (0.06 wt%) was negligible. After four cycles, the XRD pattern of the spent Pd/UiO-66-NO_2_-*7* was in accordance with the fresh catalyst, and no new crystal phase was observed (Figure S4), suggesting that the MOF structure was well maintained. The XPS spectra of fresh and spent Pd/UiO-66-NO_2_-*7* catalyst are shown in Figure 13. The XPS survey spectrum (Figure 13a) confirmed the existence of Zr, O, N, and C in the samples. The weak signal of Pd was due to the relatively low content of Pd in the catalyst. The 3d_5/2_ and 3d_3/2_ peaks of the Pd^0^ appear at 335.4 and 340.3 eV, respectively (Figure 13b). No obvious peak of Pd^2+^ is observed, indicating that palladium was in the reduced form [46]. Compared to the fresh catalyst, after the four consecutive cycles, the binding energy of Pd did not change, indicating that the Pd/UiO-66-NO_2_-*7* catalyst was not affected by the oxygen-containing reactant. The particle size distribution obtained from TEM analysis for the spent Pd/UiO-66-NO_2_-7 catalyst after four cycles is shown in Figure S5. The average particle size of Pd hardly changed after the reaction. It is therefore concluded that the decrease of activity and selectivity might be attributed to the adsorption of the polymer onto the surface of the catalyst.

## 3. Materials and Methods

### 3.1. Materials and Reagents

Zirconium tetrachloride (ZrCl_4_, 98%) and Palladium(II) chloride (PdCl_2_, 59% Pd) were purchased from J&K Scientific Co., Ltd. (Beijing, China), terephthalic acid (H_2_BDC, 98%), N,N-dimethylformamide (DMF, 99.5%), hydrofluoric acid (HF, 40%), absolute ethanol (99.7%), hexane (98%), furfural (99%), furfuryl alcohol (98.5%), tetrahydrofurfuryl alcohol (99%), cyclopentanone (97%) and cyclopentanol (99%) were purchased from Sinopharm Chemical Reagent Co., Ltd. (Shanghai, China), 2-nitro-1,4-benzenedicarboxylicacid (NO_2_-H_2_BDC, 98%) was purchased from Aladdin Industrial Inc. (Shanghai, China). Deionized water was purchased from Dalian University of Technology. All chemicals were of analytical grade and used without further purification.

### 3.2. Catalyst Preparation

Synthesis of UiO-66-NO_2_: UiO-66-NO_2_ was synthesized according to a reported procedure using minor modification [47]. Equimolar amounts (0.91 mmol) of Zirconium (IV) chloride and 2-nitro-1,4-benzenedicarboxylicacid (NO_2_-H_2_BDC) were dissolved in 50 mL of DMF, after which 39 μL hydrofluoric acid was added to the solution under vigorous stirring for 10 min at room temperature. Afterward, the resulting mixture was brought into a 150 mL pressure-resistant bottle and held at 120 °C for 24 h under static conditions. After cooling down to room temperature, the solid product was collected by centrifugation and redispersed in 50 mL of DMF solution and stirred at 80 °C for 3 h to remove the unreacted linker. The solids were separated by centrifugation and washed with ethanol via Soxhlet extraction for 24 h, followed by drying at 120 °C under vacuum. For comparison, UiO-66 was synthesized according to the same protocol, except that equivalent molar amounts of terephthalic acid (H_2_BDC) were used instead of NO_2_-H_2_BDC.

Preparation of Pd/UiO-66-NO_2_: The encapsulation of Pd nanoparticles inside the cavities of UiO-66-NO_2_ was conducted by a double-solvent method (DSM) according to a reported procedure [48]. In the DSM, hexane and water were used as the hydrophobic and hydrophilic solvent, respectively. Typically, the UiO-66-NO_2_ was activated at 120 °C for 5 h. Then, 0.1 g UiO-66-NO_2_ was added to 20 mL of anhydrous hexane and sonicated for 1 h until a homogeneous dispersion was achieved. Hereafter, 75 μL of a PdCl_2_ aqueous solution (0.6 mol·L^−1^) was added dropwise under vigorous stirring. The resulting mixture was stirred for another 2 h. The solids were separated by centrifugation and dried at 120 °C for 6 h. The solution was collected for complexometric titration of Pd to determine the Pd loading [26]. The final loading of Pd is 3 wt%, Note that all the Pd species was successfully loaded onto the MOF support by using the DSM method. The obtained Pd^2+^/UiO-66-NO_2_ sample was transferred into a U-shaped quartz reactor and reduced in an H_2_ flow (75 mL·min^−1^) at 200 °C for 4 h. After cooling down to room temperature in the presence of the H_2_ flow, the resulting catalyst was passivated in a flow of 0.5 vol.% of O_2_ in argon (20 mL·min^−1^) for 2 h before use (denoted as Pd/UiO-66-NO_2_). Pd/UiO-66 was prepared by a DSM in a similar procedure. For comparison, a UiO-66-NO_2_-supported Pd catalyst was prepared by the conventional impregnation method (denoted as Pd/UiO-66-NO_2_(IMP)). The detailed preparation procedure is described in the Supplementary Information.

### 3.3. Catalyst Characterization

Powder X-ray diffraction (XRD) patterns were collected on a Rigaku D/Max-2400 diffractometer at 40 kV and 100 mA equipped with Ni-filtered Cu-Kα radiation. N_2_ adsorption/desorption isotherms were measured on a Micrometritics Tristar II 3020 instrument at −196 °C. The sample was out-gassed under vacuum at 120 °C for 3 h prior to measurement. The specific surface area of the sample was calculated using the BET equation from the N_2_ adsorption isotherms. The elemental analysis was acquired by inductively coupled plasma optical emission spectroscopy (ICP-OES) on an Optima 2000DV device. The morphology and particle size observation of the samples were characterized by a field-emission scanning electron microscope (FE-SEM) (FEI NOVA NanoSEM 450). The pre-dried sample powder was coated with a thin layer of gold. The transmission electron microscopy (TEM) images were recorded on a Tecnai G2 F30 transmission microscope operated at 300 kV. The samples were dispersed in ethanol under ultrasonic and a drop of the suspension was deposited on a copper grid. Thermal gravimetric analysis (TG) was performed on an SDT Q600 thermogravimetric analyzer (TA Instruments, at a heating rate of 10 °C·min^−1^ from room temperature to 1000 °C in air. The X-ray photoelectron spectroscopy (XPS) measurements were performed on an ESCALAB XI^+^ X-ray photoelectron spectrometer, using an Al-Kα source. Pyridine-adsorbed FT-IR spectrum was recorded on an Equinox 55 FT-IR spectrometer. The CO chemisorption measurement was carried out using a dynamic pulse method on a Chembet-3000 instrument. The dispersion of Pd (D) and the turnover frequency (TOF) was calculated as follows:

(1) $$U_{ad}=U_{pulse}\times (n-\sum_{i}^{n}\frac{S_{i}}{S_{max}})$$

(2) $$D\;(\%)=\frac{A\times U_{ad}}{\nu \times W}$$

(3) $$\mathrm{TOF}=\frac{n_{0}\times C}{t\times \omega_{cat}\times U_{ad}}$$

where $U_{pulse}$ is the moles of CO injected each time, n is the number of pulses, $S_{i}$ is the chromatographic peak-area of each pulse, $S_{max}$ is the chromatographic peak-area of saturated adsorption, A is the atomic weight of Pd, ν is the stoichiometry factor, W is the weight of supported Pd, n_0_ is the initial moles of FAL, *C* is the conversion of FAL, *t* is the reaction time, $\omega_{cat}$ is the catalyst mass, $U_{ad}$ is the total moles of CO adsorbed on the sample.

### 3.4. Catalytic Reactions

The catalytic hydrogenative ring-rearrangement of FAL was conducted in a quartz-lined 100 mL stainless steel autoclave. In a typical run, 5.0 mg catalyst, 100.0 mg FAL, and 9.9 g water were added into the autoclave. The autoclave was purged with H_2_ to remove the residual air. After purging five times, the reactor was pressurized with H_2_ to 1.0 MPa, and then heated to a predetermined reaction temperature. The stirring speed was set at 650 rpm. At the end of the reaction, the reactor was cooled to room temperature with an ice bath. The catalyst was collected by centrifugation, and the liquid products were analyzed by GC (Agilent 6890) using an HP-INNOWax capillary column (30 m × 320 µm × 0.5 µm) using nitrogen gas as a carrier gas and an FID detector. The temperature-programmed was as follows: initial temperature of 40 °C for 3 min, increase 20 °C·min^−1^ up to 220 °C, hold 5 min. The volume of injected sample was 1 μL. The quantitative analysis was determined by the area normalization using response factors of the corresponding standard compounds. The response factors of FAL, FOL, THFOL, CPO and CPL were 0.79, 0.86, 0.88, 0.47 and 0.49, respectively. The carbon balances of all examined catalysts were in the range of 89 to 93%.

The conversion of FAL, product selectivity and yield is calculated as follows:

(4) $$\mathrm{Conversion}=(1\;-\;\frac{\mathrm{moles}\;\mathrm{of}\;\mathrm{FAL}\;\mathrm{after}\;\mathrm{reaction}}{\mathrm{moles}\;\mathrm{of}\;\mathrm{FAL}\;\mathrm{introduced}})\times 100\%$$

(5) $$\mathrm{Selectivity}=\frac{\mathrm{moles}\;\mathrm{of}\;\mathrm{each}\;\mathrm{product}}{\mathrm{sum}\;\mathrm{of}\;\mathrm{moles}\;\mathrm{of}\;\mathrm{products}}\times 100\%$$

(6) $$\mathrm{Yield}=\frac{\mathrm{moles}\;\mathrm{of}\;\mathrm{each}\;\mathrm{product}}{\mathrm{moles}\;\mathrm{of}\;\mathrm{FAL}\;\mathrm{introduced}}\;\times 100\%$$

Adsorption measurements: The adsorption of FAL onto the supports was determined as follows. Before adsorption, UiO-66-NO_2_ and UiO-66 were dried at 120 °C for 5 h. The activated MOFs (0.05 g) were added into an aqueous solution of FAL (0.3 g in 30 mL). The obtained mixture was stirred at 150 °C for 5 h. During the adsorption, aliquots from the solution were collected at regular intervals and analyzed by GC.

Hot filtration experiment: After performing the FAL hydrogenation reaction for 1 h, the catalyst was removed from the hot solution by centrifugation. The solution was allowed to react for another 4 h under the same reaction conditions.

## 4. Conclusions

In summary, the encapsulation of Pd nanoparticles inside the cavities of UiO-66-NO_2_ was successfully achieved by the double-solvent impregnation method. The Lewis acidity could be modified by creating defects upon a thermal treatment at 300 °C. In comparison to the pristine UiO-66-NO_2_, the UiO-66-NO_2_-*7* had more suitable Lewis acidity for the formation of CPO. Highly dispersed Pd nanoparticles inside the cages of UiO-66-NO_2_ and suitable Lewis acidity resulted into a high performance of the Pd/UiO-66-NO_2_ catalyst in the hydrogenation and ring-rearrangement steps. In addition, Pd/UiO-66-NO_2_ exhibited a higher activity and selectivity to CPO than Pd/UiO-66-NO_2_(IMP), which was prepared by the conventional impregnation method. Under the optimized reaction conditions (150 °C and 1.0 MPa H_2_ pressure for 5 h), 98.9% conversion of FAL and 96.6 % selectivity to CPO were achieved over Pd/UiO-66-NO_2_-*7*. Pd/UiO-66-NO_2_-*7* exhibited good stability in the recycling experiment, and the framework structure of Pd/UiO-66-NO_2_-*7* was maintained after four cycles. The hot-filtration experiment confirmed that no leaching of Pd occurred during the reaction.

## Figures and Tables

**Table 1:** The structural properties, metal dispersion, metal particle size and TOF of Pd-catalysts.

Catalyst	*S*_BET_	D ^a^	CO_uptake_	D ^b^	TOF
	(m^2^·g^−1^)	nm	(µmol·g^−1^)	(%)	(s^−1^)
UiO-66-NO_2_	735	-	-	-	-
UiO-66-NO_2_-*7*	765	-	-	-	-
Pd/UiO-66-NO_2_-*7*	563	0.9	12.9	49.8	2.9
Pd/UiO-66-NO_2_-*7*(IMP)	519	3.9	7.8	31.2	2.1
Pd/UiO-66-*7*	754	1.1	12.3	47.2	2.6

^a^ Measured based on TEM, ^b^ Calculated based on CO pulse chemisorption date.

**Table 2:** Hydrogenative ring-rearrangement of FAL to CPO over different catalysts.

Catalyst	Conv.	Sel. (%)				
	(%)	CPO	THFOL	FOL	2-CPE	Others
UiO-66	-	-	-	-	-	-
UiO-66-NO_2_	-	-	-	-	-	-
Pd/UiO-66	89.2	88.9	8.3	1.5	0.3	1.0
Pd/UiO-66-NO_2_	92.3	91.0	6.3	1.6	0.6	0.5
Pd/UiO-66(IMP)	83.7	81.4	9.1	5.6	0.8	3.1
Pd/UiO-66-NO_2_(IMP)	84.8	85.9	6.9	6.8	1.0	3.4

Reaction conditions: catalyst (5.0 mg), FAL (100.0 mg), H_2_O (9.9 g), 150 °C, 1.0 MPa H_2_ pressure, 5 h.

**Table 3:** Hydrogenative ring-rearrangement of FAL to CPO over Pd/UiO-66-NO_2_-*t* and Pd/UiO-66-*7*.

Catalyst	Conv.	Sel. (%)				
	(%)	CPO	THFOL	FOL	2-CPE	Others
Pd/UiO-66-NO_2_	92.3	91.0	6.3	1.6	0.6	0.5
Pd/UiO-66-NO_2_-*1*	94.7	94.2	4.2	1.4	-	0.2
Pd/UiO-66-NO_2_-*3*	95.8	95.1	3.6	1.2	-	0.1
Pd/UiO-66-NO_2_-*5*	98.9	96.5	2.7	0.7	-	0.1
Pd/UiO-66-NO_2_-*7*	98.9	96.6	2.5	0.6	-	0.3
Pd/UiO-66-NO_2_-*12*	98.9	95.6	2.4	-	-	2.0
Pd/UiO-66-NO_2_-*24*	98.9	92.3	2.5	-	-	5.2
Pd/UiO-66-*7*	94.2	91.6	5.9	1.6	0.2	0.7

Reaction conditions: catalyst (5.0 mg), FAL (100.0 mg), H_2_O (9.9 g), 150 °C, 1.0 MPa H_2_ pressure, 5 h.

## Data Availability

Not applicable.

## Supplementary Materials

The following are available online. Preparation of Pd/UiO-66-NO_2_(IMP); Figure S1: XRD patterns, Figure S2: SEM images, Figure S3: N_2_ adsorption/desorption isotherms, Figure S4: XRD patterns, Figure S5: TEM image; Table S1: Textural properties of the catalyst samples.
