# Supplementary material for: Hydrogenative Ring-Rearrangement of Furfural to Cyclopentanone over Pd/UiO-66-NO2 with Tunable Missing-Linker Defects
Source: Molecules. 2021 Sep 22;26(19):5736. doi: 10.3390/molecules26195736 (PMC8510385; doi:10.3390/molecules26195736)
Supplement: Supplementary file 1 [file molecules-26-05736-s001.zip › molecules-1354566-supplementary.pdf]

Supplementary Material for

Hydrogenative ring-rearrangement of furfural to cyclopentanone over Pd/UiO-66-NO_2 with tunable missing-linker defects

Chunhua Wang [1,2,†], Zhiquan Yu [1,2,†], Yuhao Yang [1,2], Zhichao Sun [1,2], Yao Wang [1,2], Chuan Shi [1], Ying-Ya Liu [1,2,*], Anjie Wang [1,2,*], Karen Leus[3] and Pascal Van Der Voort[3]

[1] State Key Laboratory of Fine Chemicals, School of Chemical Engineering, Dalian University of Technology, Dalian 116024, P. R. China ; 274638799@mail.dlut.edu.cn (C.W.); yuzhiquan@dlut.edu.cn (Z.Y.); yyh917401123@163.com (Y.Y.); sunzhichao@dlut.edu.cn (Z.S.); wangyao@dlut.edu.cn (Y.W.); chuanshi@dlut.edu.cn (C.S.)

[2] Liaoning Key Laboratory of Petrochemical Technology and Equipment, Dalian University of Technology, Dalian 116012, P. R. China；Liaoning Key Laboratory of Petrochemical Technology and Equipment, Dalian University of Technology, Dalian 116012, P. R. China; 274638799@mail.dlut.edu.cn (C.W.); yuzhiquan@dlut.edu.cn (Z.Y.); yyh917401123@163.com (Y.Y.); sunzhichao@dlut.edu.cn (Z.S.); wangyao@dlut.edu.cn (Y.W.)

[3] Department of Chemistry, Ghent University, Krijgslaan 281-S3, 9000 Ghent, Belgium; karen.leus@ugent.be (K.L.); pascal.vandervoort@ugent.be (P.V.D.V.)

[*] Correspondence: yingya.liu@dlut.edu.cn (Y.Y.L); ajwang@dlut.edu.cn (A.W.); Tel.: +86-411-84986461

[†] Equal contribution

Contents:

1. Experimental.

2. Figure S1. XRD patterns of (a) the simulated UiO-66-NO_2, pristine UiO-66-NO_2, and the UiO-66-NO_2 sample with different thermal treatment hours, (b) the simulated UiO-66, pristine UiO-66, UiO-66-*7*, and Pd/UiO-66-*7*.

3. Figure S2. SEM images of (a) UiO-66-NO_2, (b) UiO-66-NO_2-*1*, (c) UiO-66-NO_2-*3*, (d) UiO-66-NO_2-*5*, (e) UiO-66-NO_2-*7*, (f) UiO-66-NO_2-*12*, (g) UiO-66-NO_2-*24*, (h) UiO-66, (i) UiO-66-*7*, (j) Pd/UiO-66-NO_2-*7*, (k) Pd/UiO-66-*7*.

4. Figure S3. N_2 adsorption/desorption isotherms of (a) UiO-66-NO_2-*t* with different thermal treatment time, (b) UiO-66-*7*, Pd/UiO-66-*7*, UiO-66-NO_2-*7*, Pd/UiO-66-NO_2-*7*, and Pd/UiO-66-NO_2-*7*(IMP).

5. Table S1 Textural properties of the catalyst samples.

6. Figure S4. XRD patterns of fresh and spent Pd/UiO-66-NO_2-*7*.

7. Figure S5. TEM image and particle size distribution for Pd/UiO-66-NO_2-*7* after four cycles.

1. Experimental

Preparation of Pd/UiO-66-NO_2(IMP): The Pd/UiO-66-NO_2(IMP) catalyst was prepared by conventional impregnation method. 0.1 g of UiO-66-NO_2 was dispersed in 10 mL ethanol and sonicated for 30 min at room temperature. An excess amount of Pd solution was used to obtain an equal amount of Pd loading of 3 wt.%. 280 μL aqueous solution of 1.5 wt.% $PdCl_2$ in diluted HCl was dissolved in ethanol, and the Pd-containing solution was added dropwise to the UiO-66-NO_2 suspension under vigorous stirring. The resulting mixture was stirred at 50 °C for 24 h. Then the solid product was collected by centrifugation and the supernatant was collected for complexometric titration of Pd. The obtained Pd^{2+}/UiO-66-NO_2 was washed three times with ethanol, followed by drying at 120 °C under vacuum for 12 h, and then was transferred into a U-shaped quartz reactor and reduced in an H_2 flow (75 mL·min^{-1}) at 200 °C for 4 h. After cooling down to room temperature in the presence of the H_2 flow, the resulting catalyst was passivated in a flow of 0.5 vol.% of O_2 in argon (20 mL·min^{-1}) for 2 h before use (denoted as Pd/UiO-66-NO_2(IMP)).

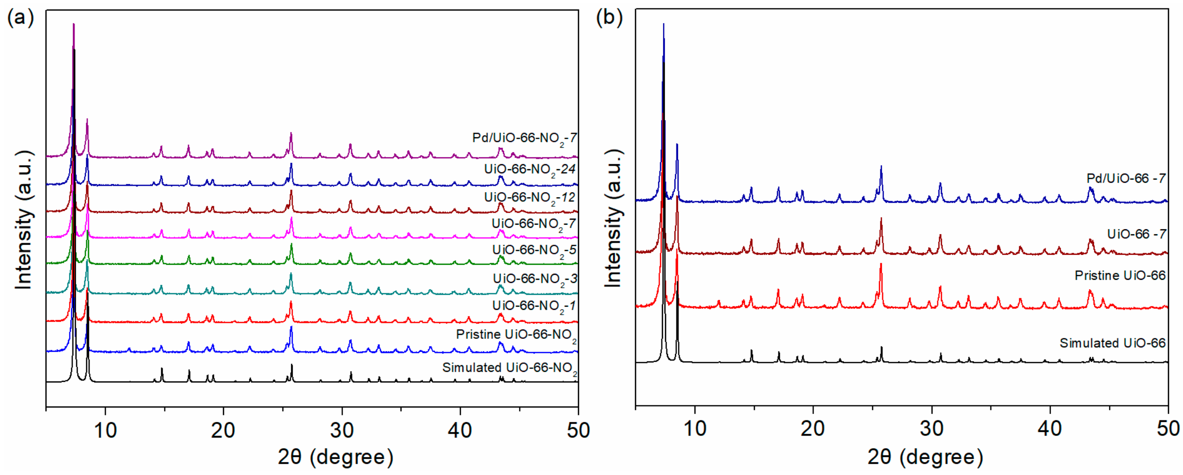

Figure S1. XRD patterns of (a) the simulated UiO-66-NO$_2$, pristine UiO-66-NO$_2$, and the UiO-66-NO$_2$ sample with different thermal treatment hours, (b) the simulated UiO-66, pristine UiO-66, UiO-66-7, and Pd/UiO-66-7.

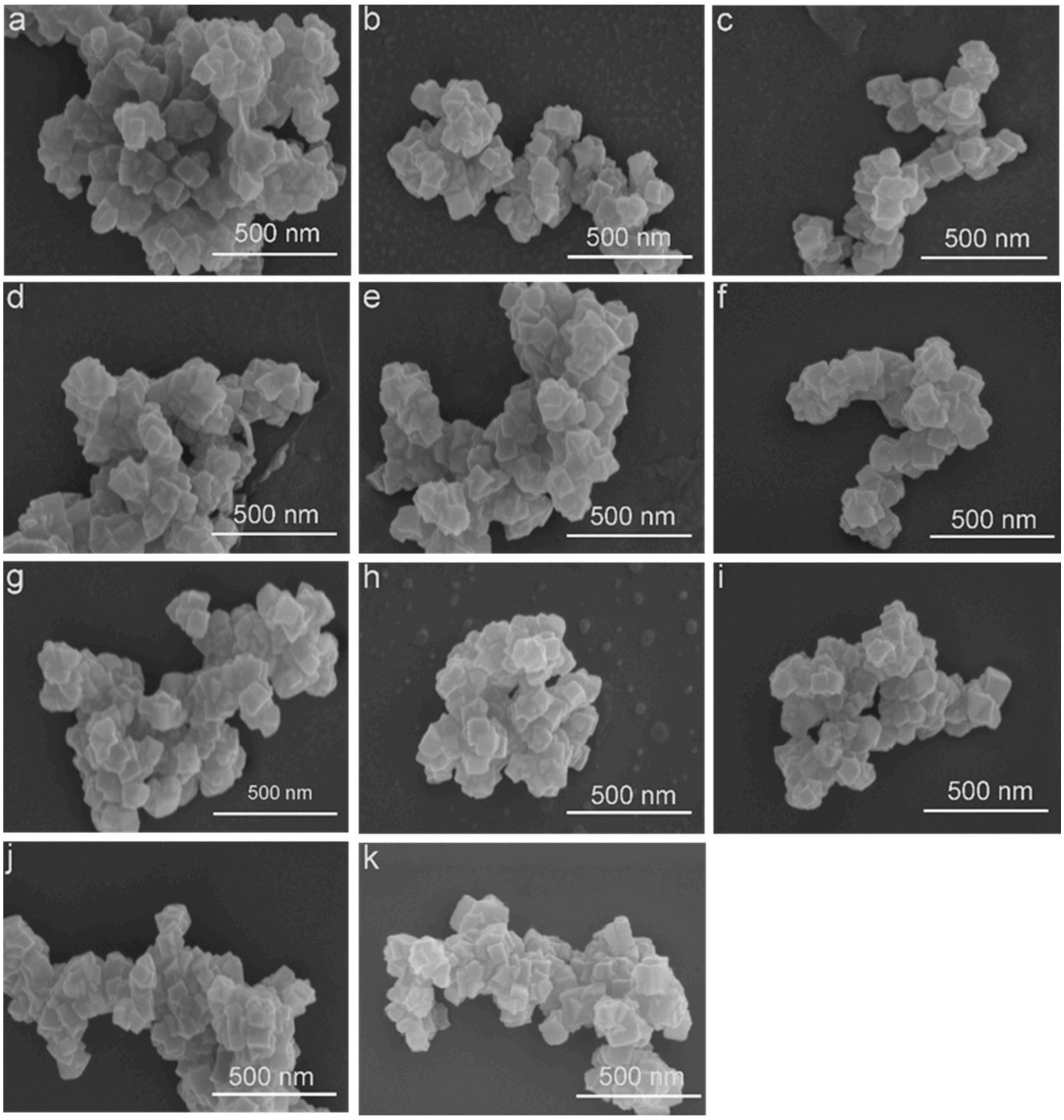

Figure S2. SEM images of (a) UiO-66-NO_2, (b) UiO-66-NO_2-*1*, (c) UiO-66-NO_2-*3*, (d) UiO-66-NO_2-*5*, (e) UiO-66-NO_2-*7*, (f) UiO-66-NO_2-*12*, (g) UiO-66-NO_2-*24*, (h) UiO-66, (i) UiO-66-*7*, (j) Pd/UiO-66-NO_2-*7*, (k) Pd/UiO-66-*7*.

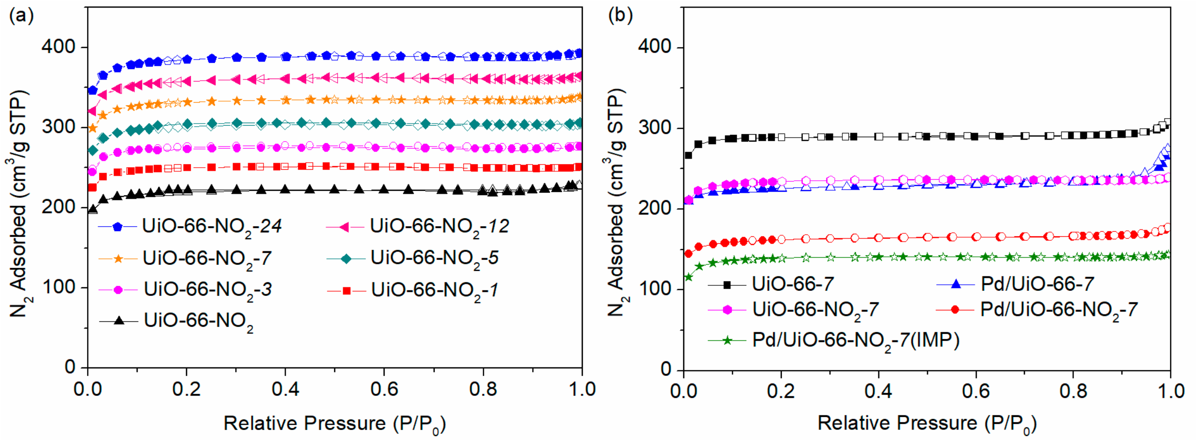

Figure S3. N_2 adsorption/desorption isotherms of (a) UiO-66-NO$_2$-*t* with different thermal treatment time, (b) UiO-66-*7*, Pd/UiO-66-*7*, UiO-66-NO$_2$-*7*, Pd/UiO-66-NO$_2$-*7*, and Pd/UiO-66-NO$_2$-*7*(IMP).

Table S1. Textural properties of the catalyst samples.

**Samples**	S_{BET} ($m^2 \cdot g^{-1}$)	V_{micro} ($cm^3 \cdot g^{-1}$)	$V_{Total\ pore}$ ($cm^3 \cdot g^{-1}$)
UiO-66-NO$_2$	735	0.25	0.35
UiO-66-NO$_2$-*1*	741	0.26	0.35
UiO-66-NO$_2$-*3*	751	0.26	0.35
UiO-66-NO$_2$-*5*	757	0.27	0.36
UiO-66-NO$_2$-*7*	784	0.28	0.37
UiO-66-NO$_2$-*12*	796	0.28	0.38
UiO-66-NO$_2$-*24*	816	0.29	0.39
Pd/UiO-66-NO$_2$-7	563	0.18	0.28
Pd/UiO-66-NO$_2$(IMP)	519	0.16	0.27

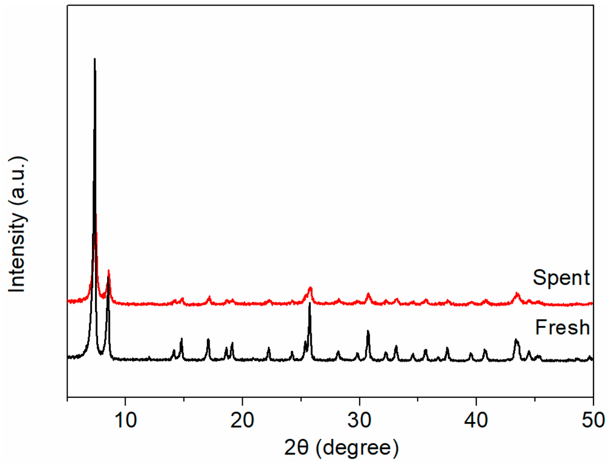

Figure S4. XRD patterns of fresh and spent Pd/UiO-66-NO_2-7.

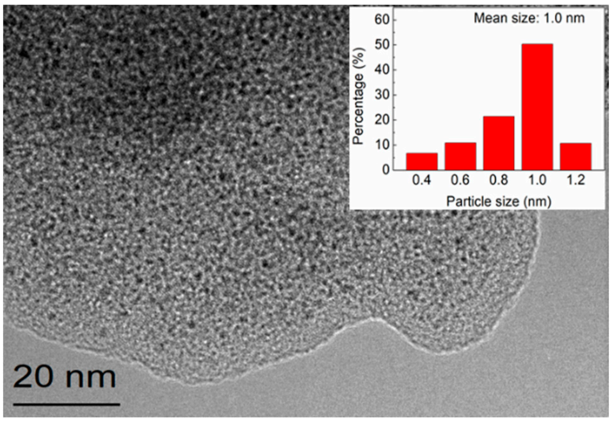

Figure S5. TEM image and particle size distribution for Pd/UiO-66-NO_2-7 after four cycles.
